# Bridging the Cultivation Gap in Plant Microbiomes: A Comparative Study of Aerial Root Mucilage Microbiome Characterization by Conventional Isolation, Prospector High‐Throughput Cultivation, and Molecular Profiling

**DOI:** 10.1002/mbo3.70268

**Published:** 2026-03-15

**Authors:** Esaú De la Vega‐Camarillo, Amanda C. Quattrone, Sakiko Okumoto, Nithya Rajan, Cesar Hernández‐Rodríguez, Julio S. Bernal, Sanjay Antony‐Babu

**Affiliations:** ^1^ Departamento de Microbiología, Laboratorio de Biología Molecular de Bacterias y Levaduras Escuela Nacional de Ciencias Biológicas México City México; ^2^ Department of Entomology Texas A&M University College Station Texas USA; ^3^ Department of Plant Pathology and Microbiology Texas A&M University College Station Texas USA; ^4^ Department of Soil and Crops Sciences Texas A&M University College Station Texas USA

**Keywords:** 16S rRNA sequencing, cultivation methods, high‐throughput isolation, methodological comparison, microbiome, Prospector IsolationBio

## Abstract

The “great plate count anomaly” represents a fundamental challenge in microbiome research, with vast microbial diversity remaining uncultivable. We systematically compared three methodological approaches for characterizing plant‐associated bacterial communities: conventional plate cultivation, the high‐throughput Prospector platform, and full‐length 16S rRNA nanopore sequencing. Using mucilage‐associated bacteria from teosinte and sorghum as model systems, we evaluated efficiency, taxonomic coverage, and inherent biases. The Prospector platform dramatically outperformed conventional cultivation, achieving 8x to 13.5x improvements in isolate recovery (342 vs. 43 isolates from sorghum; 379 vs. 28 from teosinte) and 1.5x to 1.8x improvements in genus‐level detection. While metabarcoding detected 82 total genera, cultivation methods captured only 35.4% of this diversity, with Prospector recovering 16.9%–25.7% compared to 11.3%–14.3% for conventional methods. Each approach exhibited distinct taxonomic biases: conventional plating favored fast‐growing taxa (*Pseudomonas*, *Pantoea*, *Bacillus*), Prospector accessed slower‐growing bacteria (*Sphingomonas*, *Curtobacterium*), while metabarcoding exclusively detected 59–85 cultivation‐resistant genera. We propose an integrated framework leveraging complementary strengths: metabarcoding for comprehensive profiling, Prospector for enhanced cultivation efficiency, and conventional isolation for targeted applications. Together, our findings establish quantitative benchmarks for method comparison and support an integrative framework that combines metabarcoding for comprehensive profiling, the Prospector platform for enhanced cultivation efficiency, and conventional isolation for targeted applications, highlighting how methodological choices fundamentally shape our understanding of microbial diversity.

## Graphical abstract

Integration of high‐throughput microfluidic isolation, Sanger identification, and full‐length 16S rRNA sequencing reveals a more accurate and comprehensive view of mucilage‐associated microbiomes, enabling robust cross‐validation between cultivation and community profiling.

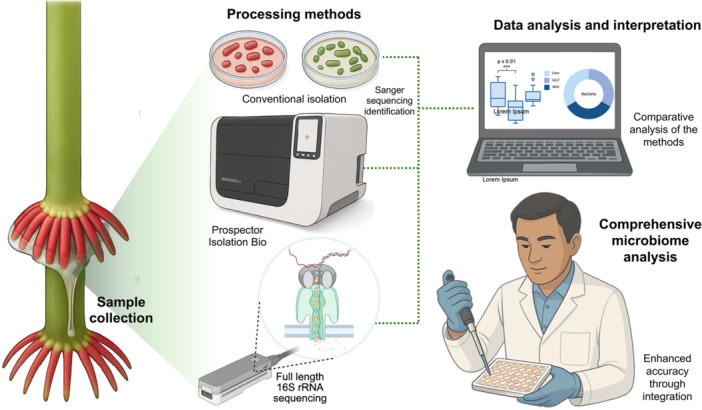

## 1 Introduction

The advent of high‐throughput molecular technologies has transformed our understanding of microbial communities through metagenomics, metatranscriptomics, and other omics approaches. However, these culture‐independent methods generate hypotheses that ultimately require cultured isolates for experimental validation. Functional characterization of predicted metabolic pathways, biotechnological exploitation of novel enzymes, and verification of ecological roles all depend on access to viable microbial cultures (Liu et al. 2022; Orsi 2023). The study of plant‐associated microbiomes has revealed their critical roles in host health, stress tolerance, and ecosystem functioning (Compant et al. 2019; Trivedi et al. 2020). However, our understanding of these complex communities remains constrained by a fundamental methodological challenge: most environmental microorganisms are not cultivable under laboratory conditions, a phenomenon known as the “great plate count anomaly” (Staley and Konopka 1985; Steen et al. 2019). This cultivation bottleneck has profound implications for microbiome research, as it limits access to microbial resources for biotechnological applications and prevents functional validation of predicted metabolic capabilities.

Traditional cultivation methods typically recover less than 1% of the microbial diversity observed through molecular approaches (Handelsman et al. 1998; Rappé and Giovannoni 2003; Bodor et al. 2020). This dramatic underrepresentation stems from multiple factors: inadequate reproduction of natural growth conditions, competitive exclusion by fast‐growing organisms, and our limited understanding of complex nutritional and signaling requirements (Stewart 2012; Kapinusova et al. 2023). The resulting bias toward easily cultivable taxa such as *Pseudomonas*, *Bacillus*, and *Enterobacter* has skewed our perception of microbial community structure and function in natural environments. Recent technological advances have begun to address these limitations through innovative cultivation strategies. High‐throughput cultivation platforms, such as the Prospector technology developed by IsolationBio Inc., employ miniaturized array‐based formats that maintain spatial segregation while testing multiple growth conditions simultaneously (Jiang et al. 2016; Persyn et al. 2022). These systems have shown promise in accessing previously uncultivable microorganisms by preventing competitive exclusion and providing diverse microenvironments that better mimic natural conditions (Lewis et al. 2021; Wu et al. 2020).

Concurrently, next‐generation sequencing (NGS) technologies have greatly widened our ability to profile microbial communities comprehensively. Full‐length 16S rRNA sequencing using third‐generation sequencing platforms like Oxford Nanopore provides species‐level resolution while avoiding amplification biases associated with short‐read technologies (Lewis et al. 2021). However, molecular methods cannot distinguish between viable and non‐viable cells, nor do they distinguish metabolically active cells from cryptobiosis or dormancy stages, directly assess functional capabilities, or provide isolates for further study (Castle et al. 2019; Galazzo et al. 2020). Furthermore, a bias in relative abundances resulting from the different content of ribosomal operons among prokaryotic species can be inferred. Despite the widespread adoption of cultivation‐based and molecular approaches in microbiome research, systematic comparisons of their relative performance remain surprisingly scarce. Most studies employ either cultivation or sequencing in isolation, missing opportunities to leverage complementary strengths (Sergaki et al. 2018). The few existing comparative studies have focused primarily on human or soil microbiomes, leaving plant‐associated communities understudied despite their agricultural and ecological importance (Pascale et al. 2020).

Plant mucilage presents an ideal model system for methodological comparison due to its defined spatial boundaries and distinct selective pressures. This polysaccharide‐rich matrix, secreted by aerial roots and other plant tissues, harbors specialized microbial communities adapted to fluctuating hydration and unique carbon sources (Nazari et al. 2022). The relatively lower diversity compared to bulk soil, combined with strong host‐specific selection, makes mucilage communities tractable for comprehensive analysis while maintaining ecological relevance.

We selected sorghum (*Sorghum bicolor*) and teosinte (*Zea mays* ssp. parviglumis) as complementary model systems to distinguish host‐specific from method‐specific effects. Teosinte, as the wild ancestor of maize, enables investigation of domestication effects on microbiome cultivability, while sorghum provides phylogenetic comparison as a related C4 grass. Both species produce abundant aerial root mucilage, facilitating consistent sampling and methodological comparison.

Here, we present a systematic evaluation of three methodological approaches for characterizing plant‐associated microbiomes: (1) conventional plate cultivation using selective media, (2) the Prospector IsolationBio high‐throughput platform, and (3) full‐length 16S rRNA nanopore sequencing. Using mucilage‐associated bacteria from two distinct host plants, we addressed the following questions: First, what are the quantitative relationships between cultivation‐based and molecular diversity estimates? How do methodological biases influence perceived community structure? Can high‐throughput cultivation technologies substantially improve access to microbial diversity? And ultimately, how can we optimize method selection and integration for comprehensive microbiome characterization? Our findings reveal method‐specific biases that fundamentally shape observed diversity patterns, demonstrate the superior performance of high‐throughput cultivation, and provide evidence‐based guidelines for designing integrated microbiome studies. This work contributes to the growing effort to bridge the cultivation gap and maximize our ability to observe and access microbial diversity for scientific and practical applications.

## 2 Materials and Methods

### 2.1 Samples Collection and Microbial Concentration Determination

Teosinte mucilage samples were obtained from mature plants growing in a self‐seeding plot established in 2014 in College Station, TX. These plots are maintained without applying pesticides or chemical fertilizers and without irrigation (i.e., are rainfed). Sorghum mucilage samples were obtained from test hybrids from Texas A&M sorghum breeding program, planted in the Brazos Bottom Experimental Field Station in College Station. Both teosinte and sorghum samples were collected in July 2024. Three biological replicates were collected for each plant species (*n* = 3 for sorghum, *n* = 3 for teosinte), with each replicate consisting of mucilage pooled from 5 to 7 individual plants. Mucilage collection was performed following the method described by Van Deynze et al. (2018), with minor modifications. Briefly, approximately 0.5 mL of mucilage was collected from aerial brace roots in the case of teosinte (Sparks et al. 2022) and crown roots in the case of sorghum using sterile forceps and immediately mixed with 4.5 mL of sterile phosphate‐buffered saline (PBS, pH 7.4) to prevent desiccation and maintain microbial viability.

The mixture was sonicated for 10 min in a CPX1800 ultrasonic bath (Fisherbrand, USA) operating at 40 kHz and 100 W to detach microorganisms from the mucilage matrix. Sonication was performed at room temperature (22°C–24°C) to maintain microbial viability. A series of tenfold serial dilutions ranging from 10⁻¹ to 10⁻⁵ was prepared to determine the concentration of microorganisms in the sample. The most probable number (MPN) technique was used with resazurin as a metabolic indicator dye, following the protocol outlined by Jarvis et al. (2010). Briefly, 100 µL of each dilution was inoculated into 96‐well microplates containing 100 µL of R2A broth (Reasoner and Geldreich 1985) supplemented with 0.02 g/L resazurin. The plates were incubated at 30°C for 48 h, and a color change from blue to pink was recorded as growth.

### 2.2 Metabarcoding Sequencing

In order to compare the cultivated bacteria in our study to the overall diversity, we performed a 16S rRNA gene sequence metabarcoding analyses. Total DNA was extracted from 0.5 mL of mucilage samples using the ZymoBIOMICS DNA Miniprep Kit (Zymo Research, USA) following the manufacturer's instructions. The full‐length 16S rRNA gene was amplified using the primers 27 F (5’‐AGAGTTTGATCMTGGCTCAG‐3’) and 1492 R (5’‐TACGGYTACCTTGTTACGACTT‐3’) (Lane 1991). PCR was performed in 25 µL reactions containing 12.5 µL of 2X KAPA HiFi HotStart ReadyMix, 0.5 µM of each primer, and 2 µL of template DNA. We performed PCR with initial denaturation at 95°C for 3 min, followed by 25 cycles of 98°C for 20 s, 55°C for 15 s, and 72°C for 30 s, with a final extension at 72°C for 5 min.

We purified PCR products using the AMPure XP system (Beckman Coulter, USA), quantified using the Qubit dsDNA HS Assay Kit (Thermo Fisher Scientific, USA), and pooled in equimolar concentrations. We synthesized the pooled library using the Oxford Nanopore Technologies (ONT) Rapid Sequencing Kit (SQK‐RAD004), allowing a streamlined library preparation process. Sequencing was performed on a MinION device using R10.4.1 flow cells, following the manufacturer's protocols for the Rapid Sequencing Kit. The sequencing run was conducted for 48 h to ensure sufficient coverage and read length.

### 2.3 Bioinformatics Analysis

Bioinformatic analysis of full‐length 16S rRNA sequences obtained from Oxford Nanopore sequencing was performed using the EzBioCloud Microbiome Taxonomic Profiling (MTP) pipeline (Yoon et al. 2017). Raw reads were filtered using NanoFilt v.2.8.0 with a minimum quality score of Q10 and a minimum length of 1400 bp. Filtered reads were processed using Porechop v.0.2.4 for adapter removal and demultiplexing.

Taxonomic assignment was performed against the EzBioCloud MTP pipeline v2.0 16S database using BLAST with the following thresholds: species (≥ 99.5%), genus (≥ 96.5%), family (≥ 92.5%), order (≥ 89.5%), class (≥ 86.5%), and phylum (≥ 83.5%) (Kim et al. 2012). The diversity analysis included both alpha and beta diversity metrics. All statistical analyses were subjected to Benjamini‐Hochberg FDR correction for multiple comparisons, with significance determined at *p* < 0.05. Community composition differences were assessed using PERMANOVA with 999 permutations through the VEGAN package.

### 2.4 Isolation by Picking From a Petri Dish

An aliquot of 100 µL from the 10^‐3^ and 10^‐4^ dilutions was spread onto Petri dishes in triplicate containing R2A agar (Difco, Detroit, MI, USA) and LGI medium (Cavalcante and Döbereiner 1988) for the isolation of oligotrophic and diazotrophic bacteria, respectively. The plates were incubated at 30°C for 48–72 h under aerobic conditions. After incubation, isolated colonies were counted, and colony‐forming units (CFU) per mL of mucilage were calculated. One representative colony from each morphologically distinct isolate was transferred to a well in a 96‐well plate containing 200 µL of R2A broth for subsequent use. The plates were incubated at 30°C for 24 h and then stored at 4°C until further analysis. Three technical replicates were used for conventional isolation.

### 2.5 Isolation Using Prospector

The Prospector technology (IsolationBio, CA, USA) was employed for the high‐throughput isolation of microorganisms, as described by Persyn et al. (2022). Prospector arrays (IsolationBio, CA, USA) containing 6,109 individual wells (41 rows × 149 columns) with 3 nL volume each. Briefly, 1.5 mL of a 1:100 mucilage suspension was mixed with 1.5 mL of R2A medium, 0.2‐0.3 cells per well (0.5–1 × 10⁵ cells/mL) for optimal single‐cell occupancy following Poisson distribution and 48 µL of fluorescent dye to load the Prospector microarray, 100 µmol/L in the loaded array. The microarray was incubated at 30°C for 24 h in a humidified chamber to prevent desiccation. Each biological replicate was processed independently through Prospector.

We monitored growth by comparing the fluorescence at t0 and t1 (24 h) using green (excitation 470 nm, emission 525 nm) and red (excitation 585 nm, emission 624 nm) filters. Fluorescence ratios were calculated to identify wells exhibiting microbial growth, with a growth threshold set at a green/red fluorescence ratio ≥ 1.5, indicating metabolic activity. Cells from positive wells were then transferred to 96‐well plates containing 200 µL of R2A broth for further assays and storage.

### 2.6 DNA Extraction and Identification

Crude DNA extracts were obtained by heat lysing the bacterial colonies. Individual colonies from the 96‐well stock plates were suspended in 20 µL of sterile molecular biology‐grade water in PCR tubes. We heated cell suspensions at 95°C for 10 min in a thermal cycler to lyse the cells, followed by centrifugation at 12,000 x *g* for 2 min. The supernatant containing the crude DNA extract was used as a template for PCR amplification. The 16S rRNA gene was amplified using the universal bacterial primers 27 F (5′‐AGAGTTTGATCMTGGCTCAG‐3’) and 1492 R (5′‐TACGGYTACCTTGTTACGACTT‐3’) (Lane 1991). PCR was performed in 25 µL reactions containing 12.5 µL of 2X KAPA HiFi HotStart ReadyMix (Roche, Switzerland), 1.25 µL of 0.5 µM of each primer, and 2 µL of template DNA. The PCR conditions were initial denaturation at 95°C for 3 min, followed by 30 cycles of 98°C for 20 s, 55°C for 15 s, and 72°C for 60 s, with a final extension at 72°C for 5 min. Forward reads were sequenced by Eton Bioscience Inc. (San Diego, CA, USA) using Sanger technology in an ABI 3730xl sequencer (Applied Biosystems, now Thermo Fisher Scientific, Waltham, MA, USA).

### 2.7 Comparative Analysis of Detection Methods

To comprehensively evaluate the performance and complementarity of bacterial detection methods, we implemented a multi‐faceted analytical framework comparing conventional cultivation, Prospector IsolationBio technology, and full‐length 16S rRNA sequencing.

#### 2.7.1 Data Standardization and Integration

Before comparative analyses, abundance data were standardized across methods following established protocols (Love et al. 2014). For cultivation‐based methods, relative abundances were calculated as the proportion of each genus relative to total isolates per method. Metabarcoding data were rarefied to 1000 valid reads per sample using the rarefy function in VEGAN v2.6‐2 (Oksanen et al. 2022) to ensure comparable diversity estimates across samples.

#### 2.7.2 Diversity Assessment and Coverage Analysis

We calculated alpha diversity indices for each method, including Shannon entropy, Simpson's diversity, and Chao1 richness estimator using the diversity function in VEGAN (Oksanen et al. 2022). Rarefaction curves were generated with the rarecurve function using 999 permutations to assess sampling completeness (Gotelli and Colwell 2001). Species accumulation curves were computed using the specaccum function with the “random” method and 999 permutations to evaluate whether additional sampling effort would yield new taxa (Colwell et al. 2012). Good's coverage estimator was calculated as C = 1 ‐ (*n*₁/*N*), where *n*₁ represents singletons and *N* represents total observations (Good 1953), providing an estimate of sampling completeness for each method.

#### 2.7.3 Method‐Specific Bias Quantification

To identify systematic biases in taxon detection, we performed differential abundance analysis using DESeq. 2 v1.34.0 (Love et al. 2014) with method type as the primary factor. Genera were classified as method‐exclusive if detected by only one approach, method‐enriched if significantly overrepresented (log₂ fold change > 2, Benjamini‐Hochberg adjusted *p* < 0.05), or method‐depleted if significantly underrepresented (log_2_ fold change <−2, adjusted *p*< 0.05). Indicator species analysis was conducted using the multipatt function in the indicspecies package v1.7.12 (De Cáceres and Legendre 2009) with 9999 permutations to identify genera most strongly associated with each detection method.

#### 2.7.4 Correlation and Community Similarity Analyses

Multiple correlation metrics were employed to assess concordance between methods (Shade et al. 2013):

Abundance‐based correlations: We calculated Spearman rank correlation coefficients for (i) all detected genera, (ii) shared genera only, and (iii) log(x + 1) transformed abundances to reduce the influence of highly abundant taxa (Pan et al. 2024). Pearson correlations were computed on centered log‐ratio (CLR) transformed data to address compositional constraints (Gloor et al. 2017).

Community structure comparisons: Procrustes analysis used the procrustes function in vegan to assess congruence in ordination space (Peres‐Neto and Jackson 2001). Mantel tests (9,999 permutations) evaluated correlations between method‐derived Bray‐Curtis distance matrices using the mantel function (Legendre and Legendre 2012). The RV coefficient was calculated as a multivariate generalization of the squared correlation coefficient using the coeffRV function in FactoMineR v2.4 (Lê et al. 2008).

Similarity indices: Binary Jaccard and Sørensen‐Dice indices assessed presence/absence patterns (Jaccard 1912; Dice 1945), while Bray‐Curtis dissimilarity quantified abundance‐based differences (Bray and Curtis 1957). All indices were calculated using the vegdist function in vegan.

#### 2.7.5 Network Analysis of Method‐Taxa Associations

Bipartite networks were constructed with detection methods and bacterial genera as two distinct node sets using the graph_from_incidence_matrix function in igraph v1.3.5 (Csárdi and Nepusz 2006). Network‐level metrics, including connectance, weighted modularity (Q), and nestedness (NODF ‐ Nested Overlap and Decreasing Fill), were calculated using the bipartite package v2.17 (Dormann et al. 2008). Genera were classified as “generalists” if detected by all methods, or as “specialists” if detected only by a single approach. Network visualization was performed using the ggbipart extension for ggplot2 (Flores et al. 2016).

#### 2.7.6 Statistical Modeling of Detection Probability

Generalized linear mixed models (GLMMs) were developed to predict detection probability using the glmer function in lme4 v1.1‐30 (Bates et al. 2015). The model structure was:

Detection~Method+log(Abundance_NGS)+(1∣Genus)

Detection was modeled as a binomial response, method as a fixed effect, metabarcoding abundance as a covariate proxy for true abundance, and genus as a random effect to account for taxon‐specific detection probabilities. Model selection was performed using AICc (Burnham and Anderson 2002).

#### 2.7.7 Performance Metrics and Method Evaluation

Composite performance metrics were calculated following Schloss et al. (2011):

- Coverage efficiency = (Genera detected/Total genera in metabarcoding) × 100
- Unique contribution = Genera detected exclusively/Total genera detected by the method
- Complementarity index = 1 ‐ (Shared genera/Union of genera across methods)

Cost‐effectiveness analysis incorporated time investment and resource requirements normalized to the genera detected, following protocols from Lagkouvardos et al. (2017).

#### 2.7.8 Bootstrap Validation and Sensitivity Analysis

All correlation analyses were subjected to bootstrap resampling (*n* = 1000) to generate 95% confidence intervals using the boot package v1.3‐28 (Canty and Ripley 2021). Sensitivity analysis evaluated metric stability across abundance thresholds ranging from 0.01% to 1% relative abundance. Robustness was assessed using leave‐one‐genus‐out cross‐validation to identify influential taxa‐driving method correlations.

#### 2.7.9 Statistical Software and Reproducibility

Analyses were performed using R v4.2.1 (R Core Team 2022) and Python v3.8.10 (Van Rossum and Drake 2009) with the following packages: NumPy v1.21.0 (Harris et al. 2020), Pandas v1.3.4 (McKinney 2010), SciPy v1.7.3 (Virtanen et al. 2020), and scikit‐learn v1.0.2 (Pedregosa et al. 2011). Statistical significance was assessed at *α* = 0.05 with Benjamini‐Hochberg false discovery rate correction for multiple comparisons (Benjamini and Hochberg 1995). All code and intermediate data files are available at https://github.com/[repository] to ensure computational reproducibility (Wilson et al. 2017).

## 3 Results

### 3.1 Detection Capacity and Method Performance Comparison

**Figure 1:**
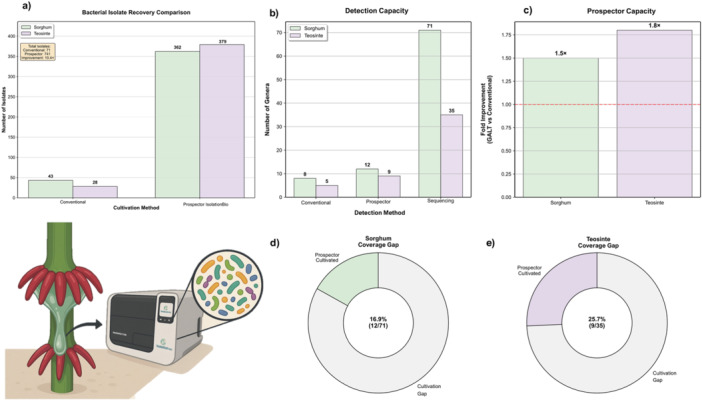
Comparative analysis of bacterial cultivation and detection methods across plant mucilage samples. (a) Total bacterial isolate recovery showing the number of individual isolates obtained from sorghum (green) and teosinte (purple) samples using conventional cultivation and Prospector IsolationBio approaches. Numerical values indicate absolute isolate counts for each method‐host combination. (b) Detection capacity comparison showing the number of distinct bacterial genera identified by conventional cultivation, Prospector, and metabarcoding (full‐length 16S rRNA sequencing) methods. (c) Prospector cultivation efficiency is expressed as fold improvement over conventional techniques. Values above bars indicate the multiplication factor of genera detected by Prospector relative to conventional approaches. The red dashed line at *y* = 1.0 represents the baseline (no improvement threshold). (d, e) Cultivation gap analysis presented as donut charts quantifying the proportion of metabarcoding‐detected bacterial diversity successfully recovered through Prospector cultivation for sorghum and teosinte, respectively. Center values show cultivation coverage percentage and absolute genera ratios (Prospector‐cultivated/metabarcoding‐detected). Colored segments represent successfully cultivated genera, while gray segments indicate the cultivation gap (genera detected by metabarcoding but not recovered through cultivation methods).

The comparative analysis revealed distinct performance profiles between cultivation methodologies across both host plants (Figure 1a). Conventional cultivation methods yielded 43 isolates from sorghum and 28 from teosinte samples, demonstrating baseline recovery capacity. The high‐throughput Prospector IsolationBio achieved substantially higher isolation efficiency, recovering 342 isolates from sorghum and 379 from teosinte samples, representing an 8x and 13.5x improvement over conventional methods.

Detection capacity analysis demonstrated a superior performance of Prospector cultivation across both the host plant species (Figure 1b). It detected eight bacterial genera for sorghum samples compared to five genera recovered through conventional cultivation methods. The performance differential was more pronounced in teosinte samples, with Prospector obtaining 12 genera versus 9 genera detected by traditional approaches. Full‐length 16S rRNA sequencing (metabarcoding) served as the reference standard, detecting 71 genera in sorghum and 35 genera in teosinte, representing the total cultivable and uncultivable bacterial diversity present in the mucilage samples.

Prospector demonstrated consistent improvement in cultivation efficiency across both host species (Figure 1c). In sorghum, Prospector achieved a 1.5x enhancement in genus detection capacity, while teosinte samples showed an even greater improvement of 1.8x compared to conventional cultivation methods. These results indicate that high‐throughput cultivation approaches can significantly expand the recoverable fraction of plant‐associated bacterial communities.

### 3.2 Cultivation Gap Analysis and Coverage Efficiency

**Figure 2:**
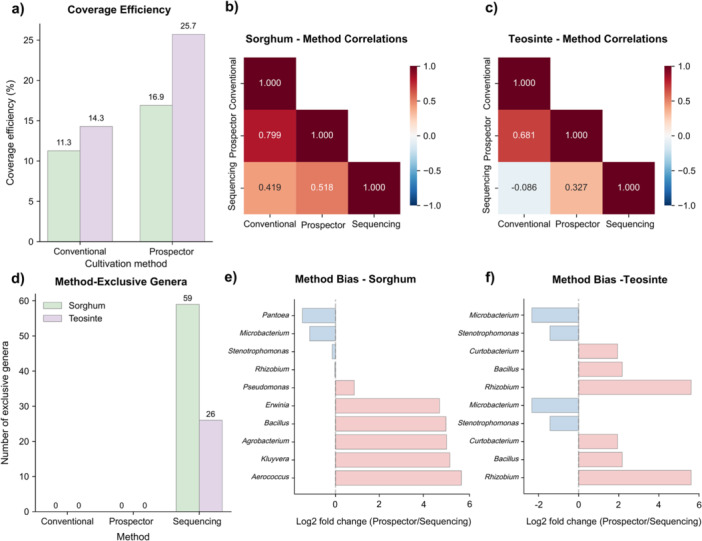
Method correlation analysis and detection bias assessment. (a) Coverage efficiency comparison between conventional cultivation and Prospector methods, expressed as percentage of total genera detected by metabarcoding. Bars are color‐coded by host plant (green for sorghum, purple for teosinte). (b, c) Spearman rank correlation heatmaps comparing genus‐level abundance profiles across the three detection methods in sorghum (b) and teosinte (c). Color intensity represents correlation strength, with correlation coefficients displayed within cells and color scale ranging from −1.0 (blue) to 1.0 (red). (d) Method‐exclusive genera count showing the number of bacterial genera detected uniquely by each platform. Note that cultivation methods detected no exclusive genera, while amplicon‐metabarcoding identified 59 and 26 exclusive genera in sorghum and teosinte, respectively. (e, f) Differential abundance analysis between Prospector and metabarcoding methods for sorghum (e) and teosinte (f), displayed as log₂ fold change values. Blue bars represent genera with higher relative abundance in Prospector compared to metabarcodes, while pink bars indicate genera with lower relative abundance in Prospector. Only genera with significant differences are shown.

Coverage efficiency analysis revealed the substantial disparity between cultivation‐based and molecular detection methods (Figure 1d‐e). In sorghum, Prospector captured only 16.9% of the total bacterial diversity detected by metabarcoding (12 out of 71 genera), leaving a cultivation gap of 83.1%. For teosinte, Prospector achieved higher relative coverage at 25.7% (9 out of 35 genera), resulting in a cultivation gap of 74.3%; the remaining portions represent the cultivation gap—bacterial taxa detected through molecular methods but not recovered through current cultivation approaches, highlighting opportunities for further methodological development. When comparing both cultivation methods across hosts (Figure 2a), Prospector consistently outperformed conventional approaches, with coverage efficiencies of 16.9% vs. 11.3% in sorghum and 25.7% vs. 14.3% in teosinte. The differential coverage efficiency between host plants indicates that teosinte mucilage harbors a higher proportion of cultivable bacteria relative to its total diversity. However, whether this reflects intrinsic differences in community composition, mucilage properties, or other host‐specific factors requires further investigation.

### 3.3 Method Concordance and Correlation Analysis

Spearman rank correlation analysis revealed varying degrees of concordance between detection methods, with host‐specific patterns of agreement (Figure 2b,c). In sorghum, PROSPECTOR showed moderate positive correlation with metabarcoding (*r* = 0.518, 95% CI [0.345, 0.650]), indicating reasonable congruence between cultivation‐based and molecular community profiles. Conventional cultivation demonstrated a weaker correlation with metabarcoding (*r* = 0.419), while the correlation between PROSPECTOR and conventional methods was strongest (*r* = 0.799), suggesting these cultivation approaches detect overlapping sets of easily culturable taxa.

For teosinte samples, method correlations were generally weaker. Prospector correlated modestly with metabarcoding (*r* = 0.327, 95% CI [−0.041, 0.607]), while conventional cultivation showed a negligible or negative correlation with metabarcoding (*r* = −0.086). The correlation between cultivation methods remained moderate (r = 0.681), confirming their tendency to recover similar taxonomic groups across different host plants.

### 3.4 Method‐Specific Detection Patterns and Exclusive Taxa

Analysis of method‐exclusive genera revealed the substantial contribution of metabarcoding to the detection of uncultivable diversity (Figure 2d). The culture‐independent assessment exclusively identified 59 genera in sorghum and 26 in teosinte that were undetectable through cultivation approaches. Notably, neither Prospector nor conventional cultivation detected any method‐exclusive genera, confirming that cultivation‐based methods primarily recover subsets of the total microbial community rather than accessing entirely distinct taxonomic groups.

Differential abundance analysis between Prospector and metabarcoding revealed systematic biases in taxon recovery (Figure 2e,f). In sorghum, genera such as *Pantoea*, *Microbacterium*, and *Stenotrophomonas* showed positive log₂ fold changes, indicating preferential detection by Prospector, while several genera including *Rhizobium*, *Pseudomonas*, and *Erwinia* were more readily detected by metabarcoding. Similar patterns were observed in teosinte, with *Microbacterium* and *Stenotrophomonas* showing PROSPECTOR bias, while *Rhizobium* and other genera were metabarcoding favored.

### 3.5 Community Structure Differences Between Plant Species

**Figure 3:**
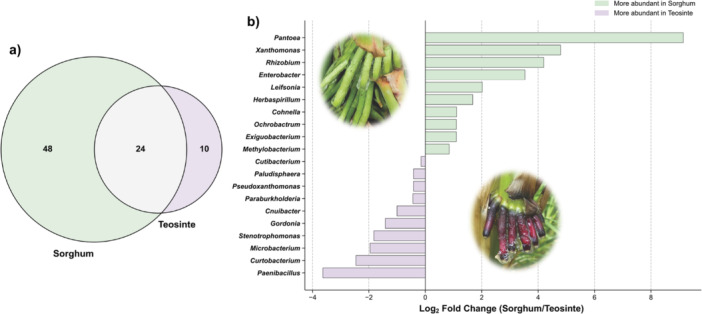
Community structure comparison between plant species. (a) Venn diagram showing the distribution of bacterial genera between sorghum and teosinte mucilage samples based on the metabarcoding data. Numbers indicate genera counts: 48 genera exclusive to sorghum, 24 genera shared between plant species, and 10 genera exclusive to teosinte. (b) Differential abundance analysis between plant species showing log₂ fold change (log₂ FC) values (sorghum/teosinte ratio). Green bars represent genera significantly more abundant in sorghum samples, while purple bars indicate genera enriched in teosinte samples. Genera are ordered by effect size magnitude. Plant photographs show representative sorghum (top) and teosinte (bottom) specimens illustrating the morphological differences between species.

Distinct patterns of bacterial genus distribution were observed between sorghum and teosinte mucilage samples (Figure 3a). Venn diagram analysis revealed 24 genera shared between both plant species, with 48 genera detected exclusively in sorghum and 10 genera detected exclusively in teosinte. Differential abundance analysis between the two plant species (Figure 3b) showed significant differences in the relative abundances of specific bacterial lineages. Sorghum samples exhibited higher log₂ fold changes for genera including *Pantoea*, *Xanthomonas*, *Rhizobium*, and *Enterobacter*, while teosinte samples showed enrichment in *Stenotrophomonas*, *Microbacterium*, *Curtobacterium*, and *Paenibacillus*.

### 3.6 Cultivated Isolate Community Composition

**Figure 4:**
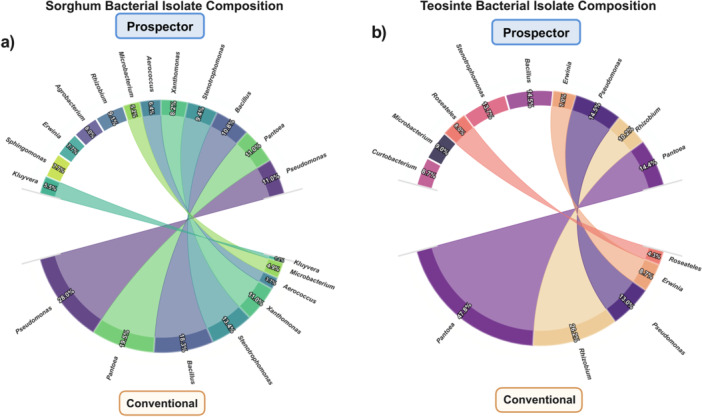
Comparative bacterial isolate composition between cultivation methods. Chord diagrams illustrating the taxonomic composition and method‐specific recovery patterns for (a) sorghum and (b) teosinte bacterial isolates. The upper semicircle represents Prospector (GALT) cultivation results, while the lower semicircle shows conventional cultivation outcomes. Chord thickness is proportional to the relative abundance of each bacterial genus within the respective cultivation method. Percentage values indicate the proportion of each genus relative to total isolates recovered by each method. Curved connections (chords) link the same genera detected by both methods, with the width reflecting the abundance in each approach. Genera appearing in only one semicircle were exclusively recovered by that cultivation method. The color scheme is consistent across both host species to facilitate comparative analysis of taxonomic patterns and methodological biases in bacterial isolate recovery.

The chord diagram analysis revealed distinct compositional patterns and methodological biases in bacterial isolate recovery across both cultivation approaches and host species (Figure 4a,b). The visualization illustrates the flow of bacterial genera between conventional and Prospector cultivation methods, with chord thickness representing the relative abundance of each genus within each cultivation approach.

In sorghum samples (Figure 4a), prospector cultivation demonstrated broader taxonomic recovery compared to conventional methods. The most abundant genera recovered through Prospector included *Pantoea* (11.0%), *Pseudomonas* (11.0%), and *Bacillus* (10.8%), while conventional cultivation was dominated by *Pseudomonas* (28.0%), *Pantoea* (19.5%), and *Bacillus* (18.3%). The chord connections reveal both shared and method‐specific genera, with *Stenotrophomonas* (9.4% Prospector vs. 13.4% conventional) and *Xanthomonas* (8.2% Prospector vs. 11.0% conventional) showing differential recovery patterns between methods.

Teosinte isolate collections (Figure 4b) exhibited even more pronounced differences between cultivation approaches. Prospector cultivation achieved more balanced genus representation, with major contributors including *Pseudomonas* (14.5%), *Bacillus* (14.5%), *Pantoea* (14.4%), and *Stenotrophomonas* (13.7%). In contrast, conventional cultivation was heavily skewed toward *Pantoea* (47.8%) and *Rhizobium* (26.2%), with limited representation of other genera. This disparity highlights the enhanced taxonomic breadth achievable through high‐throughput cultivation approaches.

The chord diagrams clearly illustrate cultivation method bias, with conventional approaches favoring select fast‐growing genera while Prospector cultivation captured broader taxonomic diversity. Several genera including *Agrobacterium*, *Erwinia*, *Kluyvera*, and *Sphingomonas*, were exclusively or predominantly recovered through Prospector in sorghum samples, while teosinte showed method‐specific recovery of *Curtobacterium*, *Roseateles*, and *Microbacterium* through Prospector cultivation. The thickness variations in chord connections demonstrate quantitative differences in genus recovery efficiency between methods.

### 3.7 Method‐Specific Detection Networks and Complementarity

**Figure 5:**
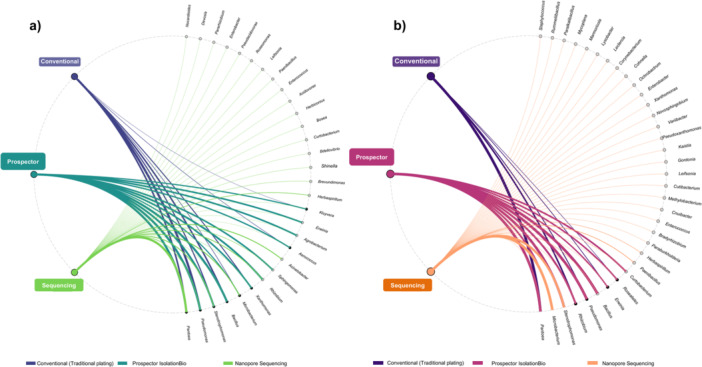
Chord diagram analysis of method‐taxa associations. Chord diagrams illustrating connections between detection methods (source nodes) and bacterial genera (target nodes) for sorghum (a) and teosinte (b) samples. Method nodes are color‐coded: dark purple for conventional cultivation, teal/magenta for Prospector, and light green/orange for metabarcoding. Chords connecting methods to genera represent successful detection, with chord width reflecting relative abundance percentages of each method‐genus association. Genera are positioned radially around the diagram perimeter and labeled. Gray nodes represent bacterial genera detected exclusively by metabarcoding.

Chord diagram analysis illustrated the complementary nature of the three detection approaches and their differential connectivity to bacterial genera (Figure 5). In sorghum (Figure 5a), the chord diagram revealed distinct detection patterns across methods, with chord width reflecting relative abundance values. Conventional cultivation was dominated by *Pseudomonas* (28.05%), *Pantoea* (19.51%), *Bacillus* (18.29%), *Stenotrophomonas* (13.42%), and *Xanthomonas* (10.98%). PROSPECTOR showed high connectivity to *Pantoea* (11.05%), *Pseudomonas* (11.05%), *Bacillus* (10.77%), *Stenotrophomonas* (9.39%), *Rhizobium* (9.12%), *Agrobacterium* (8.01%), *Xanthomonas* (8.01%), *Erwinia* (7.74%), and *Sphingomonas* (7.74%). metabarcoding connected to the broadest taxonomic spectrum, with *Pantoea* showing the highest abundance (31.16%), followed by *Microbacterium* (11.79%), *Acinetobacter* (11.12%), *Stenotrophomonas* (10.40%), *Rhizobium* (9.31%), and *Pseudomonas* (6.06%).

In teosinte (Figure 5b), conventional cultivation was dominated by *Pantoea* (47.83%), *Rhizobium* (26.09%), *Pseudomonas* (13.04%), *Erwinia* (8.70%), and *Roseateles* (4.35%). Prospector showed strong connections to *Bacillus* (14.46%), *Pantoea* (14.46%), *Pseudomonas* (14.46%), *Stenotrophomonas* (13.72%), *Microbacterium* (8.98%), *Curtobacterium* (8.73%), *Roseateles* (7.98%), *Erwinia* (6.98%), and *Rhizobium* (10.22%). Metabarcoding revealed *Microbacterium* as the most abundant genus (45.09%), followed by *Stenotrophomonas* (36.74%), *Paenibacillus* (7.63%), *Herbaspirillum* (3.72%), and *Bacillus* (3.2%).

Shared genera across all three methods in sorghum included *Pantoea*, *Pseudomonas*, *Stenotrophomonas*, and *Xanthomonas*, while in teosinte, only *Pantoea*, *Pseudomonas*, and *Rhizobium* were detected by all methods. Metabarcoding detected genera that were undetectable through cultivation approaches, including *Herbaspirillum*, *Acinetobacter*, *Brevundimonas*, *Shinella*, among others in sorghum, and *Bradyrhizobium*, *Kaistia*, *Variibacter*, among others in teosinte.

## 4 Discussion

### 4.1 Methodological Performance and Cultivation Enhancement

Our study demonstrates Prospector as an efficient and pragmatic platform for rapid and high‐throughput isolation of microorganisms from plant‐associated environments. We achieved eightfold to 13.5‐fold improvements in isolation efficiency compared to conventional cultivation methods, successfully recovering 379 unique isolates from sorghum (43 from conventional and 336 from Prospector) and 583 from teosinte (64 from conventional and 519 from Prospector) (Figure 1a). This enhancement translates to meaningful gains in genus‐level detection capacity, with Prospector achieving 1.5‐fold and 1.8‐fold improvements over conventional methods in sorghum and teosinte, respectively (Figure 1c). Our systematic comparison of cultivation‐based and molecular detection methods reveals the substantial impact of technological advancement on bridging the cultivation bottleneck, providing researchers with a scalable solution for accessing the cultivable fraction of plant microbiomes.

The cultivable fraction was substantially higher than expected, with cultivation methods capturing 16.9% to 25.7% of the metabarcoding‐determined diversity in plant‐associated environments. While metabarcoding detected 71 genera in sorghum and 35 in teosinte, Prospector cultivation captured 16.9% and 25.7% of this diversity, respectively, compared to only 11.3% and 14.3% achieved by conventional methods (Figure 1d‐e). These coverage efficiencies represent a significant advance over the typically reported < 1% cultivation success rates (Amann et al. 1995; Vartoukian et al. 2010), demonstrating that technological innovation can substantially expand access to environmental bacteria under standard laboratory conditions. The superior performance of Prospector likely stems from its capacity to maintain spatial isolation of individual cells while providing diverse microenvironments that approximate natural conditions, thereby preventing competitive exclusion by fast‐growing organisms, a long‐understood fundamental limitation of traditional plating approaches (Nichols et al. 2010; Stewart 2012).

#### 4.1.1 Method Concordance and Validation Framework

Correlation analysis revealed varying degrees of concordance between detection methods, with important host‐specific patterns (Figure 2b,c). In sorghum, Prospector showed moderate positive correlation with the amplicon metabarcoding data (*r* = 0.518), indicating reasonable congruence between cultivation‐based and molecular community profiles, while conventional cultivation demonstrated a weaker correlation with amplicon data (*r* = 0.419). The strongest correlation observed was between Prospector and traditional methods (*r* = 0.799), confirming that these cultivation approaches detect overlapping sets of easily culturable taxa, with Prospector simply recovering more diverse representatives from these taxonomic groups.

For teosinte samples, method correlations were generally weaker, with Prospector showing a modest correlation with metabarcoding (*r* = 0.327) and conventional cultivation showing a negligible correlation (*r* = −0.086). These patterns align with previous comparative studies in diverse microbial habitats (Bent and Forney 2008; Schloss & Handelsman 2005), suggesting broader applicability of our findings across different plant‐microbe systems.

The coverage efficiency comparison (Figure 2a) demonstrates Prospector's consistent superiority over conventional cultivation across both host species, with coverage efficiencies of 16.9% vs. 11.3% in sorghum and 25.7% vs. 14.3% in teosinte. Analysis of method‐exclusive genera (Figure 2d) revealed the substantial contribution of metabarcoding in detecting uncultivable diversity, with metabarcodes exclusively identifying 59 genera in sorghum and 26 in teosinte that were undetectable through cultivation approaches. Notably, neither Prospector nor conventional cultivation detected any method‐exclusive genera, confirming that cultivation‐based methods primarily recover subsets of the total microbial community rather than accessing entirely distinct taxonomic groups.

Differential abundance analysis between Prospector and the metabarcode sequences (Figure 2e,f) revealed systematic biases in taxon recovery. In sorghum, genera such as *Pantoea*, *Microbacterium*, and *Stenotrophomonas* showed positive log₂ fold changes, indicating preferential detection by Prospector, while several genera including *Rhizobium*, *Pseudomonas*, and *Erwinia* were more readily detected by metabarcoding. Similar patterns were observed in teosinte, with *Microbacterium* and *Stenotrophomonas* favoring a Prospector bias, while *Rhizobium* and other genera were better detected by culture independent amplicon metabarcoding.

#### 4.1.2 Host‐Specific Community Structure and Cultivation Efficiency

The differential cultivation efficiency between host species provides important ecological insights. Community structure analysis (Figure 3a) revealed distinct patterns of bacterial genus distribution between sorghum and teosinte mucilage samples, with 24 genera shared between both plant species, 48 genera detected exclusively in sorghum, and 10 genera detected exclusively in teosinte. Differential abundance analysis between the two plant species (Figure 3b) showed significant differences in the relative abundances of specific bacterial lineages, with sorghum samples exhibiting higher log₂ fold changes for genera including *Pantoea*, *Xanthomonas*, *Rhizobium*, and *Enterobacter*, while teosinte samples showed enrichment in *Stenotrophomonas*, *Microbacterium*, *Curtobacterium*, and *Paenibacillus*.

Teosinte mucilage consistently showed higher cultivation success (25.7% vs. 16.9% for Prospector), suggesting that teosinte harbors a higher proportion of cultivable bacteria relative to its total diversity. This pattern may reflect distinct evolutionary pressures or community assembly processes that favor more readily cultivable taxa in teosinte‐associated microbiomes compared to sorghum (Bulgarelli et al. 2013; Edwards et al. 2015). The disparity between host species, combined with the higher cultivation success in teosinte, suggests fundamental differences in the ecological strategies of bacteria associated with these closely related plant species. A wider systematic study on mucilage microbiome is perhaps warranted. Several mechanisms likely underlie these host‐specific differences in cultivation efficiency. Primarily, the variations in mucilage exudate composition between sorghum and teosinte may differentially support bacterial growth under laboratory conditions. In addition, the two hosts originate from distinct geographical regions, with teosinte evolving in Mexico and sorghum originating in northeastern Africa, which likely shaped the pools of microorganisms available for recruitment into the mucilage microbiome. Additionally, crop domestication has fundamentally altered sorghum's microbiome, potentially shifting from diverse oligotrophic communities toward copiotrophic assemblages better adapted to agricultural nutrient regimes (Pérez‐Jaramillo et al. 2016). This might explain the reduced proportion of readily cultivable bacteria in sorghum compared to wild teosinte. Comparative studies of mucilage microbiota across teosinte, landraces, and modern cultivars will be required to directly test these hypotheses. Additionally, the higher cultivation success in teosinte (25.7% vs. 16.9%) further suggests a greater prevalence of fast‐growing, nutrient‐responsive bacteria that thrive under standard cultivation conditions (Fierer et al. 2007).

#### 4.1.3 Taxonomic Composition and Method‐Specific Biases

The chord diagram analysis (Figure 4) provides critical insights into the taxonomic biases inherent in different cultivation approaches. In sorghum (Figure 4a), conventional cultivation showed extreme bias toward select genera, with *Pseudomonas* (28.0%), *Pantoea* (19.5%), and *Bacillus* (18.3%) dominating isolate collections. Prospector cultivation achieved more balanced genus representation, with the same dominant genera present at lower, more equitable proportions (*Pantoea* 11.0%, *Pseudomonas* 11.0%, *Bacillus* 10.8%). This pattern was even more pronounced in teosinte (Figure 4b), where conventional cultivation was heavily skewed toward *Pantoea* (47.8%) and *Rhizobium* (26.2%), while Prospector achieved more taxonomically diverse recovery with major contributors including *Pseudomonas* (14.5%), *Bacillus* (14.5%), *Pantoea* (14.4%), and *Stenotrophomonas* (13.7%).

The visualization of genus‐specific flows between cultivation methods clearly demonstrates that Prospector not only recovers more isolates overall but also accesses taxonomic groups that remain undetected by conventional approaches. Several genera, including Agrobacterium, Erwinia, Kluyvera, and Sphingomonas in sorghum, and Curtobacterium, Roseateles, and Microbacterium in teosinte, were exclusively or predominantly recovered through Prospector cultivation. This expanded taxonomic breadth has essential implications for functional studies and biotechnological applications that depend on accessing diverse bacterial capabilities (Davis et al. 2005; Janssen et al. 2002).

#### 4.1.4 Integrated Methodological Framework

Our bipartite network analysis (Figure 5) illuminates the complementary nature of different detection approaches, showing that while cultivation methods access only a subset of total diversity, this subset often includes ecologically or functionally important taxa. The chord plot structure reiterates that the culture‐independent method provides unique access to a significant fraction of cultivation‐resistant community members while cultivation methods share connections to overlapping cultivable genera. In sorghum (Figure 5a), the plot shows high connectivity between cultivation methods and a core set of easily culturable genera. At the same time, metabarcoding is connected to a much broader taxonomic spectrum, including numerous cultivation‐resistant taxa. The plot structure for teosinte (Figure 5b) displayed similar patterns but with reduced overall complexity, reflecting the lower total diversity in this host.

This complementarity strongly supports adopting integrated approaches that leverage the distinct strengths of each method. Amplicon metabarcoding profiles provide a comprehensive community profiling essential for understanding microbial diversity and ecological relationships, while cultivation methods yield viable isolates necessary for functional characterization, mechanistic studies, and biotechnological applications (Bai et al. 2015; Mendes et al. 2013). The moderate correlations between Prospector and metabarcoding suggest that cultivation data can provide meaningful biological insights when properly contextualized within broader community structure revealed by molecular methods.

#### 4.1.5 Method Selection Guidelines and Future Directions

Based on our comparative analysis, we propose evidence‐based suggestions for method selection in plant microbiome studies. As expected, culture‐independent molecular approaches still hold a upper‐hand in accessing diversity, providing the most comprehensive solution for diversity assessment objectives. They offer broader taxonomic coverage with minimal bias when employing full‐length 16S rRNA sequencing (Johnson et al. 2019; Wemheuer et al. 2020). When functional studies requiring viable isolates are the primary objective, Prospector or similar high‐throughput platforms should be prioritized due to their demonstrated 50%–80% improvement in genus detection over conventional methods.

For comprehensive microbiome characterization requiring community structure assessment and functional validation capabilities, integrated frameworks combining culture‐independent community profiling with enhanced cultivation approaches provide optimal coverage. This integration captures the diversity breadth revealed by molecular methods and the functional potential accessible through cultivation‐based approaches (Hassani et al. 2018; Reinhold‐Hurek et al. 2015).

#### 4.1.6 Broader Implications and Future Research

Our quantitative framework for method comparison provides standardized metrics that could be applied across diverse microbial systems, offering benchmarks for method evaluation in other microbiome contexts. The demonstration that technological innovation can meaningfully reduce, though not eliminate, the cultivation gap suggests that continued investment in cultivation methodology development remains worthwhile for the microbiology community (Kaeberlein et al. 2002; Zengler et al. 2002).

This integrative framework can be readily adapted to other plant‐microbe systems and diverse microbial ecosystems. For plant‐associated compartments such as the rhizosphere, phyllosphere, and endosphere, the framework can be tailored by adjusting cultivation conditions to match niche‐specific requirements (e.g., oxygen levels, pH, nutrient composition) and selecting molecular profiling methods appropriate to each habitat's complexity. The framework extends beyond plant systems to soil, marine, and host‐associated microbiomes, where cultivation success rates and method correlations can serve as quantitative benchmarks for evaluating isolation strategies. Key adaptations include adjusting statistical thresholds based on community complexity (e.g., lower expected cultivation efficiency in highly diverse soil communities vs. more defined symbiotic systems) and balancing molecular profiling breadth and cultivation depth according to research objectives (Lagkouvardos et al. 2017; Steen et al. 2019).

Future research should focus on expanding cultivation success to the substantial fraction of community members that remain recalcitrant to laboratory growth conditions. This might involve developing specialized media formulations, co‐cultivation approaches, or novel cultivation technologies that better approximate natural environmental conditions (D'Onofrio et al. 2010; Kato et al. 2018). Additionally, developing correction factors that account for methodological biases in comparative analyses will improve the integration of cultivation and molecular data in comprehensive microbiome studies.

Establishing correlation benchmarks between cultivation and molecular methods provides a foundation for evaluating the representativeness of cultivation‐based findings and developing integrated analytical frameworks that leverage the complementary strengths of different methodological approaches (Caporaso et al. 2010; Kato et al. 2018). As microbiome research evolves toward mechanistic understanding and practical applications, such integrated approaches will become increasingly essential for bridging the gap between community characterization and functional validation (Berg et al. 2020; Vorholt 2012).

## 5 Conclusion

This systematic comparison demonstrates that high‐throughput cultivation technologies substantially improve microbial isolation efficiency, with Prospector achieving eightfold to 13.5‐fold increases in isolate recovery compared to conventional methods. While metabarcoding provided the most comprehensive taxonomic coverage (71 genera in sorghum, 35 in teosinte), cultivation methods accessed functionally important subsets with coverage efficiencies of 16.9%–25.7% for Prospector and 11.3%–14.3% for conventional cultivation. Methodological biases significantly influenced perceived community structure, with conventional cultivation showing extreme taxonomic skewing while high‐throughput platforms achieved more balanced representation. Moderate correlations between Prospector and metabarcoding (*r* = 0.327–0.518) indicate that enhanced cultivation can provide meaningful biological insights, though substantial cultivation‐resistant diversity remains exclusively detectable by molecular methods. We recommend objective‐driven method selection: metabarcoding for comprehensive diversity assessment, high‐throughput cultivation for functional studies requiring viable isolates, and integrated frameworks for complete microbiome characterization. These quantitative metrics provide standardized benchmarks for method evaluation and support continued cultivation technology development to reduce the cultivation gap in microbiome research.

## Author Contributions

**Esaú De la Vega‐Camarillo:** conceptualization, methodology, investigation, formal analysis, data curation, visualization, writing – original draft. **Amanda C. Quattrone:** methodology, investigation, validation, formal analysis. **Sakiko Okumoto:** resources, supervision, writing – review and editing. **Nithya Rajan:** resources, supervision, writing – review and editing. **Cesar Hernández‐Rodríguez:** supervision, project administration, funding acquisition, writing – review and editing. **Julio S. Bernal:** conceptualization, resources, supervision, project administration, funding acquisition, writing – review and editing. **Sanjay Antony‐Babu:** conceptualization, methodology, resources, supervision, project administration, funding acquisition, writing – review and editing. All authors contributed to the article and approved the submitted version.

## Ethics Statement

The authors have nothing to report.

## Conflicts of Interest

The authors declare that the research was conducted without any commercial or financial relationships that could be construed as a potential conflict of interest.

## Policy on Using ChatGPT and Similar AI Tools

Artificial intelligence was used to assist in generating illustrations and debugging bioinformatics scripts. The conceptual illustration showing mucilage secretion from adventitious roots and microbial analysis via the automated bacterial isolation system (Prospector, Isolation Bio) was generated using DALL·E (OpenAI, 2025), based on detailed prompts and visual references provided by the authors. Additionally, ChatGPT (OpenAI, 2025) was used to assist in writing and refining the scripts employed for bioinformatics analyses, ensuring good practices in data processing and visualization. All AI‐generated content was reviewed, validated, and, where necessary, edited by the authors to ensure scientific accuracy and relevance.

## Data Availability

The data supporting the findings of this study are openly available at 10.6084/m9. figshare.31014802 under the title “Mucilage_microbiome.”
